# Supplementary material for: VaxArray immunoassay for the multiplexed quantification of poliovirus D-antigen
Source: J Immunol Methods. 2022 May;504:None. doi: 10.1016/j.jim.2022.113259 (PMC9072286; doi:10.1016/j.jim.2022.113259)
Supplement: Supplementary file 1 — Supplementary material [file mmc1.docx]

**SUPPLEMENTAL INFORMATION**

**Oral Poliovirus Reactivity**

Oral Poliovirus Vaccine (OPV) WHO international standards for OPV1, 2, and 3 were obtained from NIBSC (16/196, 15/296, and 16/202) with stock concentrations ranging from 10^6^-10^7^ TCID_50_/mL. Based on literature, the concentrations in terms of D-Ag/mL are expected to be from ~0.3 to 3 D-Ag/mL.^1,2^ A 10-fold dilution series for each serotype was analyzed monovalently (n=1 each dilution due to limited sample), with the highest concentration samples not diluted in PBB to enable testing at maximum concentration. A 6-hr antigen incubation time was utilized to ensure adequate signal from these low concentration samples. A linear fit was applied to each response curve to assess linearity, with the error bars expressed as ± 1 standard deviation of the median fluorescence signal. For OPV1, the highest concentration standard was omitted from the linear fit due to fluorescence saturation. We note that InDevR is a polio essential facility (PEF) in accordance with the US National Authority for Containment of Poliovirus (NAC) housed at the US Centers for Disease Control and Prevention (Atlanta, GA).

**Figure S1** shows the median signals generated by each serotype. Due to limited antigen material, a single replicate was run for each concentration with error bars representing ± 1 standard deviation of the 9 replicate microarray spots. In **Figure S1a**, the highest concentration sample for serotype 1 was fluorescently saturated and therefore not included in the fit. All three serotypes demonstrated a robust linear response curve, with R^2^ values of 0.95, 0.99, and 0.94 for T(1), T(2), and T(3), respectively. These results demonstrate that the assay can be applied to quantify OPV samples of sufficient concentration with the appropriate standards. Given that infectivity, such as TCID_50_, is required as a release assay for this live-attenuated vaccine, it is unlikely that the VaxArray Polio Assay will be a suitable release assay for OPV, but such a rapid method has utility during bioprocess development and optimization to provide complementary, actionable information with a same day turnaround time.


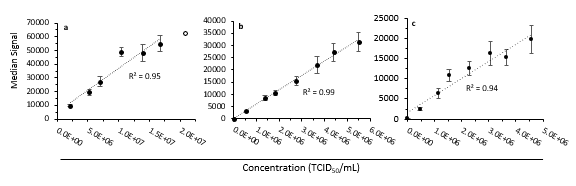


**Figure S1** Monovalent OPV response curves for (a) T(1), (b) T(2), and (c) T(3). Y-axis is median fluorescence signal generated, and linear fits to the response curves for each serotype are shown as dotted lines with the associated correlation coefficients (R^2^) indicated. In (a), the highest concentration point is omitted from the fit due to fluorescence saturation. Because a single replicate of each sample was analyzed, error bars represent ± 1 standard deviation of the 9 replicate microarray spots for each capture.

**sIPV Time to Result Reduction Studies**

To further minimize the assay time to result, additional testing was performed using the ArrayMax orbital shaker (VX-6212, InDevR, Inc.) instead of a standard orbital shaker during antigen incubation and detection labeling. Monovalent sIPV materials were combined to 100/50/100 D-Ag/mL starting concentrations for types 1/2/3 and used to create a standard curve of 15 dilutions down to 0.03 D-Ag/mL which was analyzed in triplicate. Slides were placed on the ArrayMax platform, 45 µL of samples added to the microarrays, and shaken at 700 rpm for 15 min. Samples were then removed, and 45 µL of detection label was added and allowed to shake at 700 rpm for an additional 15 min. Slides were then processed and imaged as previously described.

While the VaxArray Polio Assay was initially developed for use with a standard large orbit (20 mm diameter) shaker (such as SCI-O180-S, Scilogex) with a 3-hour time to result, here we demonstrate that it is quite feasible to obtain essentially equivalent results in under 1 hour for the VaxArray Polio Assay using a small orbit (1mm) shaker (ArrayMax, InDevR, Inc.) to improve mass transport. For the assay performed with the large-orbit shaker, 2 hr antigen incubation and 30 min labeling steps were used (2.5 hours overall on shaker). For the assay performed using the small-orbit shaker, 15 min antigen incubation and 15 min labeling steps were used (30 minutes on shaker). **Figure S2** shows comparative response curves for both the large-orbit shaker (solid lines), and the small-orbit ArrayMax shaker (dotted lines), with the smoothed fits intended only to guide the eye.


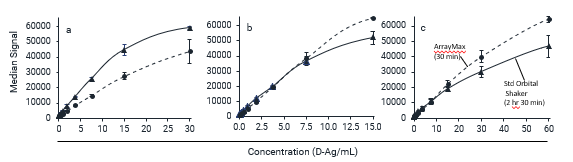


**Figure S2** Comparison of response curves using standard orbital shaker and ArrayMax orbital shaker to improve mass transport and reduce assay time to result. T(1), T(2), and T(3) are shown in panels (a), (b), and (c), respectively. Standard orbital shaker with 2-hour antigen incubation and 30-min detection labeling times is shown as filled triangles with a solid black line to guide the eye, and ArrayMax orbital shaker with 15-minute antigen incubation and 15-min detection labeling times is shown as the filled circles with the dotted line to guide the eye. Error bars shown are ± 1 standard deviation of 3 measurements. Both datasets were collected using the same 100 ms exposure time.

A similar signal response (either slightly lower or higher than the standard assay) was generated for all 3 serotypes while reducing the overall incubation time by 5x, and the same exposure time was utilized during fluorescence imaging for both methods. In addition, the error bars shown in **Figure S2** (± 1 standard deviation of the mean of 3 independent experiments) illustrate that similar precision is obtained for both methods. Achieving a rapid time to result is of particular importance for bioprocess samples in which a rapid turnaround time is desirable to produce actionable information that can inform bioprocess improvements or raise alerts during bioprocess monitoring. While the analyses conducted here were on purified poliovirus monovalent bulk vaccine materials, we have demonstrated for other VaxArray product lines (data not shown) that ArrayMax can be utilized to reduce the time to result for crude samples as well with similar results.

**References**

1. Beale, A.J., Mason, P.J. The Measurement of the D-Antigen in Poliovirus Preparations, J Hyg, 60, 113 (1962).
2. Wilton, T., Dunn, G., Eastwood, D., Minor, P.D., Martin, J. Effect of Formaldehyde Inactivation on Poliovirus, J Virol, 88 (20), 11955-11964 (2014).
